# Evolution of copper resistance in *Xanthomonas euvesicatoria* pv. *perforans* population

**DOI:** 10.1128/msystems.01427-24

**Published:** 2024-11-25

**Authors:** Amandeep Kaur, Gerald V. Minsavage, Neha Potnis, Jeffrey B. Jones, Erica M. Goss

**Affiliations:** 1 Department of Plant Pathology, University of Florida, Gainesville, Florida, USA; 2 Department of Entomology and Plant Pathology, Auburn University, Auburn, Alabama, USA; 3 Emerging Pathogens Institute, University of Florida, Gainesville, Florida, USA; Universidad Miguel Hernandez de Elche Departamento de Produccion Vegetal y Microbiologia, San Juan de Alicante, Spain

**Keywords:** antimicrobial resistance, *Xanthomonas*, genomic islands, evolution, disease management

## Abstract

The widespread use of antimicrobials that target bacterial pathogens has driven evolution of resistance, compromising the efficacy of these bactericides. Understanding the emergence and spread of resistance genes via mobile genetic elements is crucial for combating antimicrobial resistance. Copper resistance (CuR) in *Xanthomonas euvesicatoria* pv. *perforans* has severely affected the efficacy of copper-based bactericides for controlling bacterial leaf spot disease of tomato and pepper. Here, we investigated the evolutionary pathways of CuR acquisition and dissemination in *X. euvesicatoria* pv. *perforans* using an extensive collection of strains. We determined that chromosomally encoded CuR predominates over plasmid-borne CuR in multiple distinct phylogenetic groups of *X. euvesicatoria* pv. *perforans*. Our analysis revealed a single site of chromosomal integration by a CuR genomic island, although the genomic island showed sequence variation among phylogenetic groups. While chromosomal CuR was more prevalent, strains with plasmid-borne resistance conferred greater copper tolerance. Additionally, we identified strains carrying two copies of CuR genes, on plasmid and chromosome, that exhibited increased copper tolerance. Strains of *X. euvesicatoria* pv. *perforans* from the USA shared identical CuR gene sequences whether on plasmids or chromosome while different alleles were found in strains from other countries. In contrast to *X. euvesicatoria* pv. *perforans*, plasmid-borne CuR predominated in closely related pathovar, *X. euvesicatoria* pv. *euvesicatoria*. Overall, these findings contribute to a better understanding of the evolution and persistence of CuR in *X. euvesicatoria* pv. *perforans* and its closest relatives.

**IMPORTANCE:**

The emergence of antimicrobial resistance is a significant threat to agricultural production as it reduces the efficacy of various antimicrobials including copper-based bactericides that are widely used to control plant diseases. The challenge of increasing antimicrobial resistance entering a production system necessitates a deeper understanding of the dynamics and mechanisms by which pathogens acquire resistance. As a result of this research, we have identified different mechanisms of copper resistance acquisition as well as levels of copper resistance in a devastating plant pathogen, *X. euvesicatoria* pv. *perforans*. The evolution and dissemination of copper resistance in strains through plasmid or chromosomally integrated genomic island or both presents barriers to current management approaches, where growers rely heavily on copper-based bactericides to manage disease outbreaks. This knowledge is crucial when considering the continued use of existing antimicrobials or adopting alternative antimicrobials in efforts to implement enhanced antimicrobial stewardship strategies in agriculture.

## INTRODUCTION

Bacterial pathogens constantly evolve to adapt to variable environments. The evolutionary success of bacteria is shaped by their ability to respond to various selective forces, such as ecological factors and host-pathogen interactions. Antimicrobial resistance is one of the best demonstrated examples of bacterial adaptation to their environment. The rapid emergence of antimicrobial resistance on a global scale is a premier threat to modern agriculture (1, 2). While antimicrobials are widely used to control major bacterial plant diseases, their extensive use has resulted in widespread antimicrobial resistance (3, 4). To counteract the spread of antimicrobial resistance, it is important to understand the underlying evolutionary mechanisms that drive the emergence and dissemination of resistance traits.

Copper-based antimicrobials have been effectively used for years to control many plant diseases, including bacterial spot disease (BST) of tomato and pepper. In the early 1950s, streptomycin was the primary antibiotic used to manage BST in the field. However, the emergence of streptomycin-resistant strains reduced its efficacy, and growers shifted to copper-based bactericides (5). For many decades, copper-based compounds were used to control BST. The heavy usage of these compounds also resulted in the prevalence of copper resistance (CuR) in BST-causing bacterial pathogens (6). BST is caused by four *Xanthomonas* species: *X. euvesicatoria* pv. *euvesicatoria, X. euvesicatoria* pv. *perforans, X. vesicatoria,* and *X. hortorum* pv. *gardneri* (7). Among these, *X. euvesicatoria* pv. *perforans* has become the dominant pathogen of tomato species over the last three decades (8, 9). It can cause severe yield losses in transplants or field production areas under optimum conditions (10). The widespread occurrence of CuR in *X. euvesicatoria* pv. *perforans* is a serious concern, with an increasing number of copper-resistant strains being isolated worldwide (11–16). A greenhouse and field study showed the variable efficacy of copper-based formulations along with other pesticides against *X. euvesicatoria* pv. *perforans* in production fields (13). Furthermore, a field survey in Florida showed nearly complete CuR resistance in *X. euvesicatoria* pv. *perforans* strains (17). Though other alternatives, such as cultural practices, use of bacteriophages (18, 19), or various nanomaterials (20–22), are effective approaches for BST control, copper continues to be a choice for many growers and has been shown to reduce BST severity when used in combination with other molecules (13, 23, 24).

The mechanism for CuR was initially identified to be associated with a *cop* operon present on a self-transmissible plasmid in *Pseudomonas syringae* pv. *tomato* and *Xanthomonas campestris* pv. *vesicatoria* (25–27). Plasmid-mediated CuR was subsequently characterized in other *Xanthomonas* species, such as *X. campestris* pv. *campestris*, *X. citri* pv. *citri* and *X. alfalfae* subsp. *citrumelonis* (now *X. euvesicatoria* pv. *citrumelonis*), and *X. hortorum* pv. *gardneri* (28–30). The *cop* operon shares a similar gene structure across different genera; however, differences in nucleotide sequences indicate that the copper resistance genes (*cop* genes) have independently evolved among species or genera (30–34). For example, in *X. citri* pv. *citri,* the *cop* operon composition is *copLABMGCDF,* whereas in *X. alfalfae* pv. *citrumelonis,* it is *copLABMGF* (29). A recently described *cop* cluster in *X. campestris* pv. *campestris* strain BrA1 is distinct from the others reported from *Xanthomonas* (28). Within the *cop* operon, the *copLAB* genes are essential for CuR (29). The *copA* and *copB* genes code for copper-binding proteins, while *copL* is involved in regulating the expression of these genes. Other genes in the operon code for: CopM, a cytochrome *c* oxidase involved in electron transport; CopG, a hypothetical export protein; CopC and CopD, transmembrane transporters; and CopF, a copper-transporting p-type ATPase (29, 35). In addition to the *copLAB* genes, copper homeostasis genes *cohL*, *cohA*, and *cohB* have also been identified on the chromosome and are responsible for maintaining an adequate concentration of copper in the bacterial cytoplasm (36, 37).

Interestingly, CuR was not confined to plasmids, as chromosomally encoded CuR was reported in the early 1990s in *Pseudomonas syringae* and *X. campestris* pv. *juglandis* (38, 39). The horizontal transfer of chromosomal *cop* genes through conjugation was first shown in *X. axonopodis* pv. *vesicatoria* strain XvP26 (now classified as *X. euvesicatoria* pv. *euvesicatoria*) (40). In a follow-up study, a 7,652-bp region carrying *cop* genes on the chromosome was characterized in XvP26 (41). Recent studies revealed the presence of a chromosomally encoded *cop* operon on a genomic island (GEI) in *X. euvesicatoria* pv. *perforans* and *X. campestris* pv. *campestris*, highlighting the role of genomic islands in chromosomal CuR (42, 43).

Plasmids are autonomously replicating extrachromosomal genetic elements and often considered as common drivers of antimicrobial resistance gene transfer. In contrast, GEIs are integrated chromosomal DNA segments that also play a crucial role in bacterial survival and adaptation via disseminating genes related to virulence, resistance, metabolism, toxin production, or symbiosis (44, 45). The spread of CuR through diverse MGEs, including plasmids and genomic islands, raised the question of the evolution of these two forms of CuR in bacterial spot pathogens *X. euvesticatoria* pv. *euvesicatoria* and *X. euvesicatoria* pv. *perforans*. Given the potential advantage of the presence of *cop* genes on GEIs over the maintenance of a large plasmid, we hypothesized that we would see a transition from plasmid-borne to chromosomally encoded CuR in these pathogens and evidence for multiple acquisitions of CuR across their phylogenies.

To address the above hypothesis, we analyzed 452 *X*. *euvesicatoria* pv. *perforans* genomes from diverse locations to examine the distribution of CuR, whether encoded on plasmid or chromosome. The widespread presence of chromosomally encoded copper resistance, in multiple phylogenetic groups, suggests selection for chromosomally encoded CuR over plasmid-borne resistance in *X. euvesicatoria* pv. *perforans* but not in *X. euvesicatoria* pv. *euvesicatoria*. Interestingly, some strains carry two copies of CuR genes, one on the plasmid and one on the chromosome with slightly increased tolerance to copper. The examination of integration sites, core, and variable gene content of the chromosomally encoded genomic island revealed different variants of the genomic island within *X. euvesicatoria* pv. *perforans* and between pathovars but identical CuR gene variants among all *X. euvesicatoria* pv. *perforans* strains from the USA. Altogether, our study sheds light on the mechanisms of the evolution of copper resistance and its persistence in *X. euvesicatoria* pathovars.

## RESULTS

### Distribution of chromosomal and plasmid-borne CuR

To investigate the distribution of plasmid or chromosomal-encoded CuR, we analyzed 452 *X*. *euvesicatoria* pv. *perforans* genomes, available on NCBI, representing diverse geographical locations: USA (427), Australia (10), Canada (7), Turkey (6), Italy (1), and Mauritius (1). Table S1 provides metadata for the strains used in this study. Initially, all genomes were screened for the presence of CuR plasmid using the mob-suite plasmid prediction tool (46). Out of 452 genomes, 102 showed the presence of a large plasmid (~230 kb) containing *cop* genes. In 59 genomes, the *cop* genes were assembled on an 87 kb plasmid, which is 97% identical with 78% query coverage to the 230 kb plasmid. For the presence of *cop* genes on the chromosome, we performed screening for the genomic island previously reported by Bibi et al. (42). The chromosomal genomic island containing *cop* genes was identified in 264 genomes, while 27 genomes did not contain *cop* genes. The genomic island is flanked by a Tn3 transposase gene at one end and a gene coding for a phage-like protein at the other end (Table S2). The analysis of upstream and downstream sequences revealed a single integration site of the genomic island near a gene that codes for an AraC family transcriptional regulator in 143 strains, where the island was assembled in a single contig. In other strains, determination was challenging due to contig breaks in the assemblies at either the upstream or downstream end.

To confirm our *in silico* predictions from draft genomes of an 87 kb plasmid containing the *cop* operon and to ensure it was not a genomic island, we performed Nanopore sequencing and plasmid profiling of two strains, GEV1001 and GEV2048 (Fig. S1). Fully assembled genomes confirmed the presence of an 87 kb copper resistance plasmid. The complete genomes also showed that these two strains have *cop* genes on the chromosome, indicating that they carry two copies of the *cop* operon: one on the plasmid and one on the chromosome.

**Fig 1:**
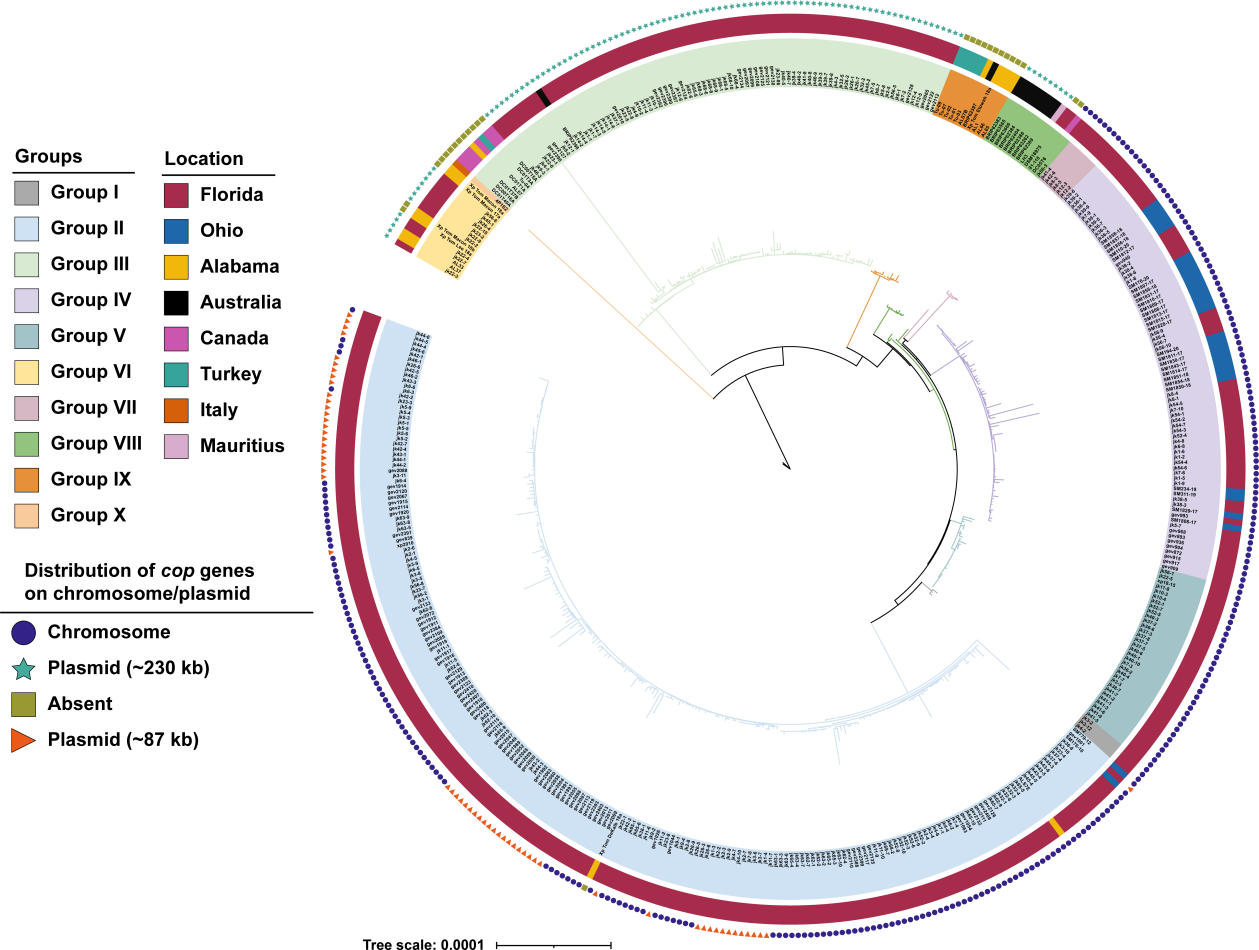
Distribution of chromosomal or plasmid-borne copper resistance in *Xanthomonas euvesicatoria* pv. *perforans* populations showing distribution of chromosomal copper resistance across multiple phylogenetic groups. The core-genome-based phylogenetic tree was constructed using PhyML and visualized using iTOL software. Background colors represent different groups as determined by fastbaps analysis. The innermost circle represents geographical location of strains, and outermost circle refers to the presence of copper genes on the genomic island or on the 230 or 87 kb plasmid, or their absence. The 230 kb copper plasmid is found in groups III and VI, while the 87 kb plasmid is present in group II strains. Notably, two strains, GEV1001 and GEV2048 from group II, which carry the 87 kb plasmid, also harbor the chromosomal genomic island.

To assess the phylogenetic relatedness of strains, a core genome phylogeny was constructed. The distribution of plasmid or chromosomal CuR was then mapped across different phylogenetic groups (Fig. 1). Fastbaps (47) analysis of core genomes identified 10 groups within the sample. Groups III, VI, and VIII contained the *cop* genes on a plasmid, while chromosomal resistance predominated in other groups. Some strains in group II carried the *cop* genes on the 87 kb plasmid and may have both plasmid-borne and chromosomal CuR like GEV1001 and GEV2048. There were genomes of strains from groups II, III, VI, VIII, and IX in which *cop* genes were not found. Note that the *copA* gene in the genomic island shows 74% nucleotide similarity to the chromosomal homologous gene *cohA*, thus they are easily distinguished.

### Higher copper tolerance in strains carrying both chromosomal and plasmid CuR

**Fig 2:**
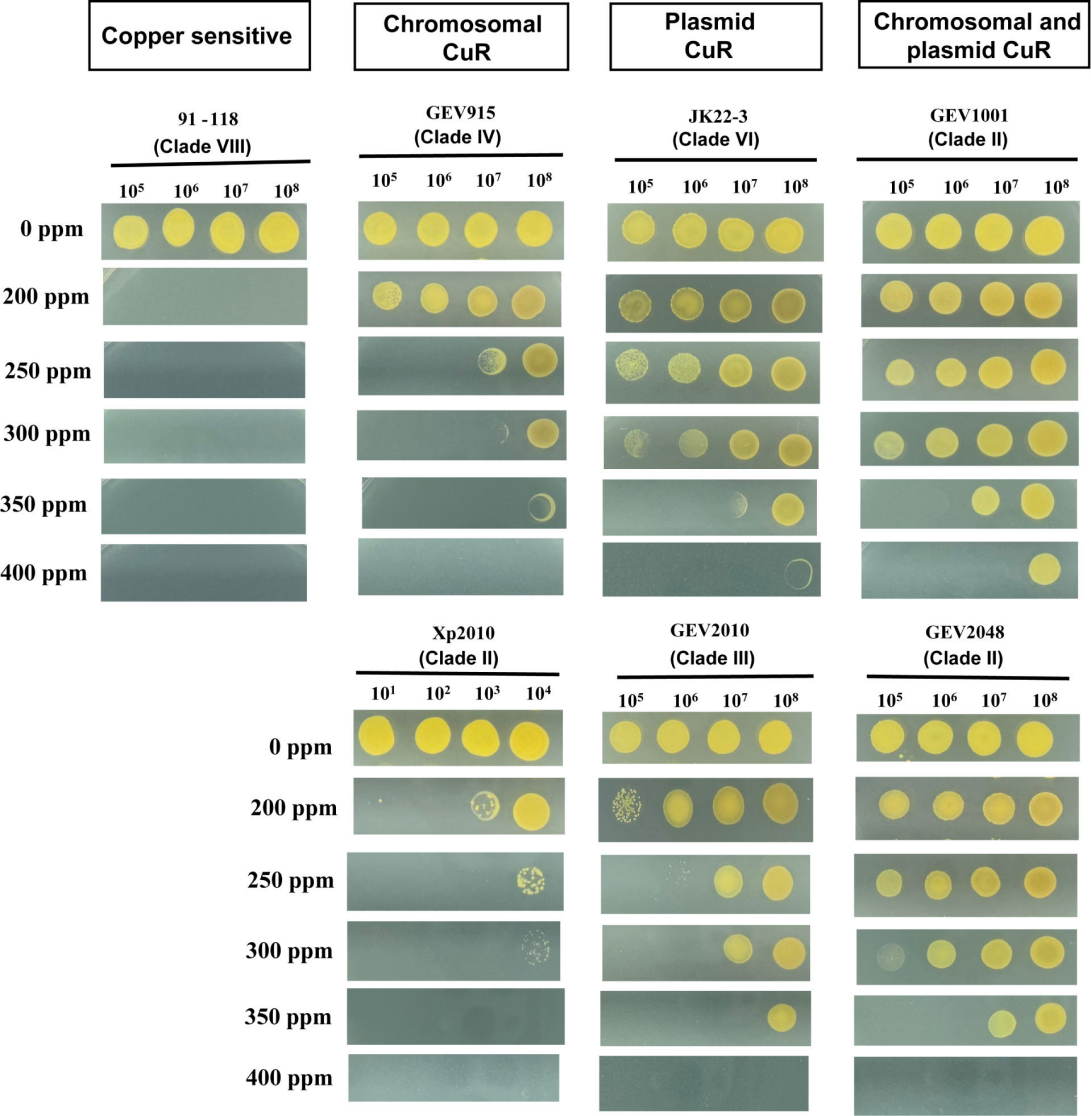
Growth of *X. euvesicatoria* pv. *perforans* strains with copper resistance genes on plasmid, chromosome, or both on nutrient agar plates amended with different concentrations of copper (200, 250, 300, 350, 400 ppm). Strain 91-118 (group VIII) was used as a negative control. Strains GEV1001 and GEV2048 (group II) have two copies of the *cop* operon, one on the 87 kb plasmid and one on the chromosome. Strains JK22-3 (group VI) and GEV2010 (group III) have the *cop* operon present only on the 230 kb plasmid, while strains GEV915 (group IV) and Xp2010 (group II) have *cop* operon present on the chromosomal genomic island. Strains with plasmid-borne copper resistance exhibited higher copper tolerance compared to those with chromosomal copper resistance.

To compare the levels of copper tolerance in *X. euvesicatoria* pv. *perforans* strains carrying chromosomal CuR, plasmid CuR, or both, we selected two strains from each category and assessed the growth at different concentrations of copper. We observed that, in general, strains carrying the *cop* operon on a plasmid exhibited greater copper tolerance compared to those with chromosomal CuR. Strains GEV1001 and GEV2048, which carry two copies of the *cop* operon, could tolerate slightly higher copper levels compared to strains JK22-3 and GEV2010 with only plasmid CuR (Fig. 2). Specifically, strains GEV1001 and GEV2048 showed higher growth compared to JK22-3 and GEV2010 when 10^7^ cfu/mL cell suspension was spotted on nutrient agar amended with 350 and 400 ppm copper. Xp2010 and GEV915, with chromosomal CuR, did not grow at these concentrations (Fig. 2).

### Different variants of genomic island across phylogenetic groups

**Fig 3:**
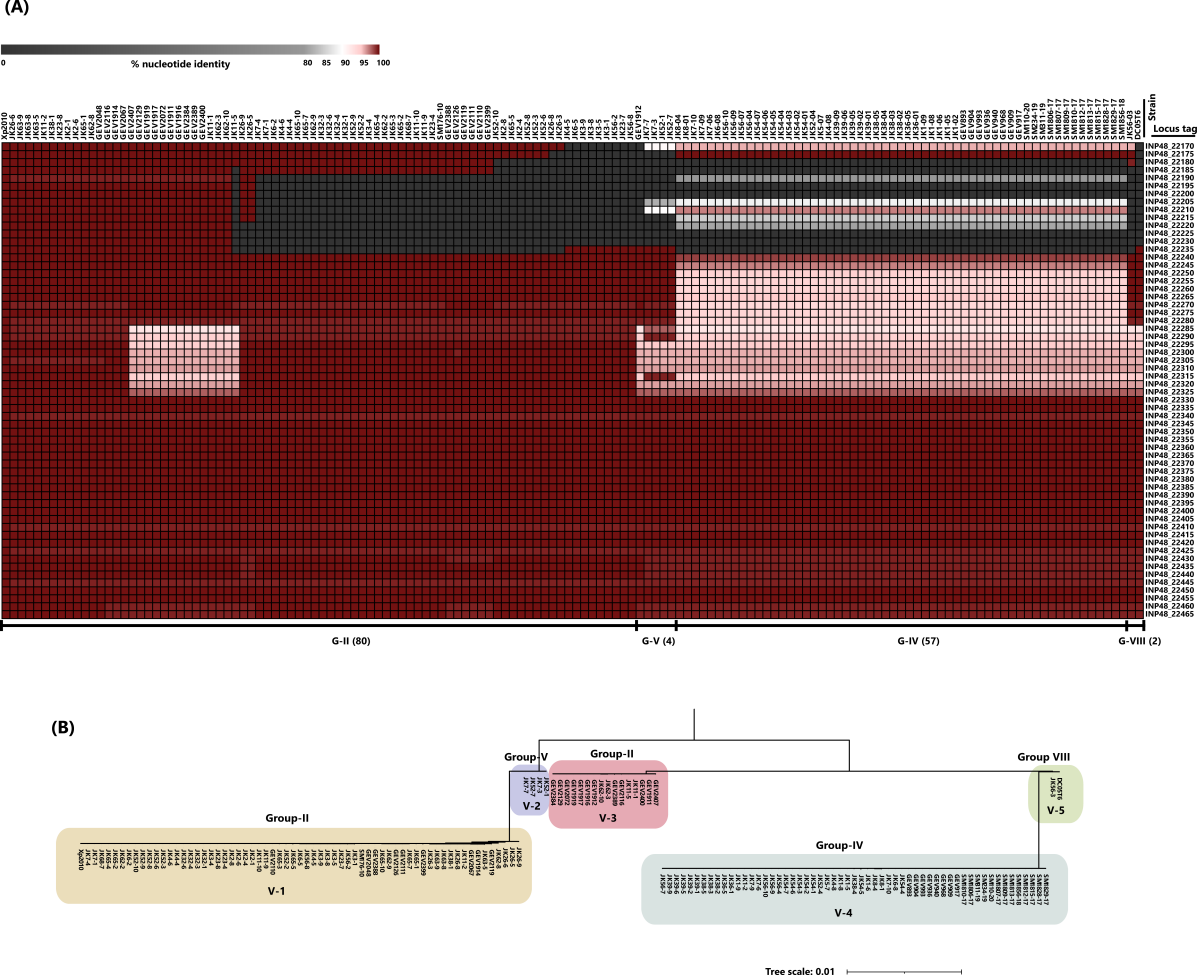
(**A**) Heatmap representing percent nucleotide identity in gene-by-gene comparisons for genes present in the genomic island to strain Xp2010 as the reference. Genes are presented at the right with their respective locus tags. Representative strains, in which the genomic island was assembled in a single contig, are shown on the top. The assignment of strains to groups based on core genome is shown below, with the total number of strains indicated in the brackets. Analysis revealed variations in the genomic island both within and between phylogenetic groups, represented by gene loss and variable nucleotide sequence identity. (**B**) Maximum likelihood phylogenetic tree constructed based on the core region (~57 kb) of the genomic island in representative strains. The five variants of the genomic island (V1–V5) are highlighted with background colors.

As chromosomal CuR was widespread across different phylogenetic groups, we checked the conservation of the genomic island. We performed BLASTN comparison using the previously identified genomic island in *X. euvesicatoria* pv. *perforans* strain Xp2010 as a reference. Gene-by-gene comparison for all 60 genes present on the island, with 70% nucleotide identity and 40% gene length coverage cutoff, was performed for all representative strains possessing the genomic island (Fig. 3A). Representative strains were selected based on the criterion that the island was assembled in one contig to avoid any false hits or interpretations that may arise due to contig breaks. In addition to *cop* genes, the genomic island contains phage genes, genes with regulatory functions, transport-related genes, and genes with unknown functions. The complete list of genes present in the genomic island is provided in Table S2. Notably, our analysis revealed variations in the genomic island both within and between phylogenetic groups, including gene loss and variable nucleotide sequence identity (ranging from 79% to 100%) (Fig. 3A). In general, the island carries a variable region (~7.3 kb) (INP48_22170 to INP48_22235) that is subjected to gene loss and constitutes genes with signature sequences typical of a genomic island, such as phage genes and hypothetical genes (Table S2). The core region (~57 kb) (INP48_22240 to INP48_22465), which includes genes present in all strains, displays variation in nucleotide sequence identity and is enriched for heavy metal resistance genes, regulatory systems, and potential efflux pumps (Table S2). The percent nucleotide identity values for all genes compared with reference strain Xp2010 are shown in Table S3.

To assess the phylogenetic relationship among variants of the genomic island, we constructed a maximum likelihood phylogenetic tree based on a multiple sequence alignment of the core region of the island (Fig. 3B). The resulting tree topology indicated five major variants within the population. All variants were specific to groups, such as variants V-1 and V-3 in group II, and V-2, V-4, and V-5 restricted to group V, IV, and VII, respectively. Importantly, gene synteny in the core region of the genomic island, including the *cop* operon, was conserved among representative strains from each variant of the genomic island (Fig. S2).

Within the core part of the island, variation was localized to specific genes: INP48_22240 to INP48_22325 in group IV and INP48_22285 to INP48_22325 in other groups. These regions include genes that code for a transcriptional regulator, heavy metal-associated proteins, proteins involved in efflux pump systems (RND [resistance-nodulation-division family transporters]), and TonB-dependent receptors. ClonalFrameML analysis on the core region of the genomic island revealed a signal of recombination (Fig. S3).

### Mobility of chromosomal-encoded CuR genomic islands

**Fig 4:**
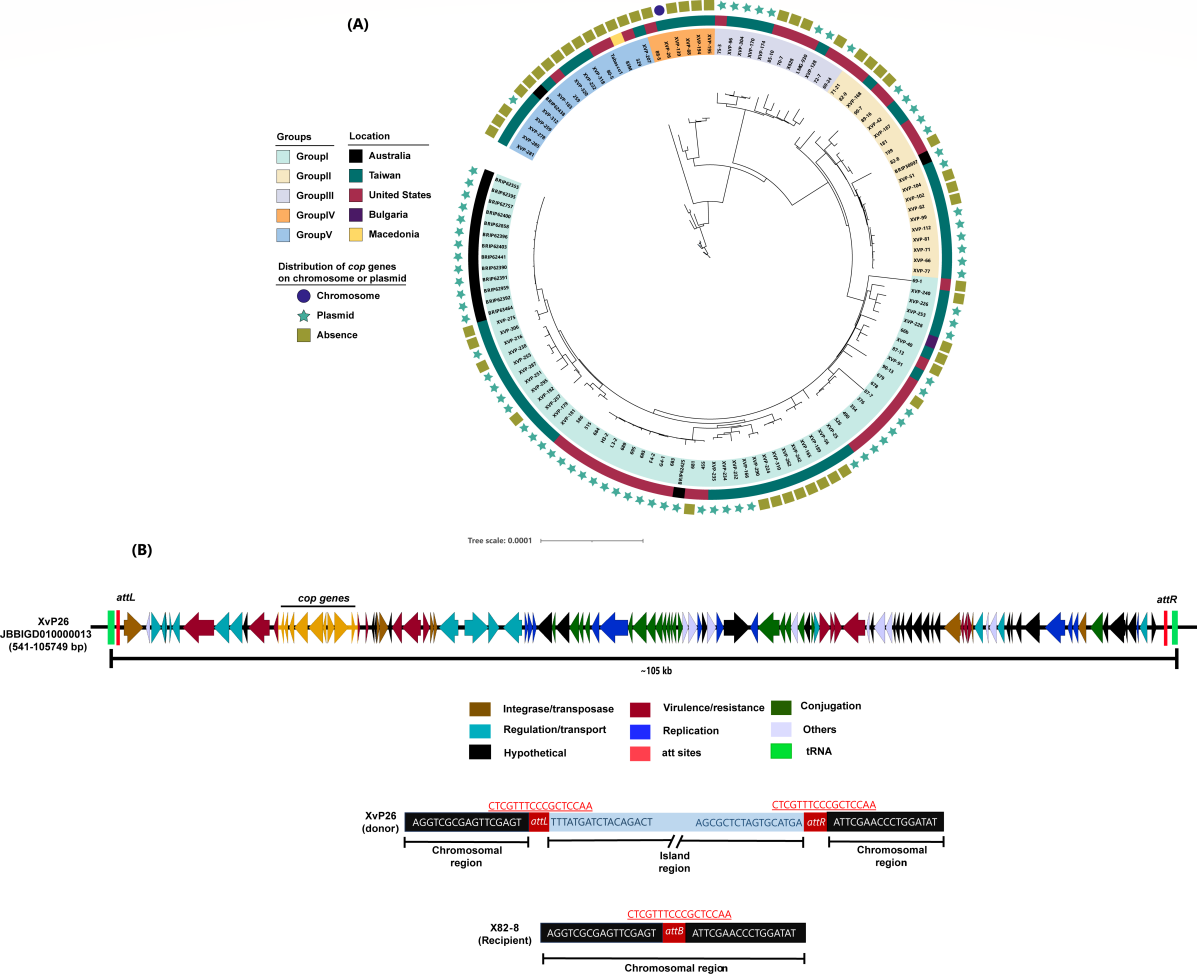
(**A**) Core-genome-based phylogenetic tree of *X. euvesicatoria* pv. *euvesicatoria* strains constructed using PhyML and visualized using iTOL software. Background colors represent different groups as determined by fastbaps analysis. The innermost circle represents geographical location of strains, and outermost circle refers to the presence of copper genes on the genomic island or plasmid or their absence. Plasmid-borne copper resistance is predominant in *X. euvesicatoria* pv. e*uvesicatoria* strains, except for strain XvP26, which carries chromosomal copper resistance. (**B**) Schematic organization of the ICE in *X. euvesicatoria* pv. *euvesicatoria* strain XvP26. ORFs with different functions are colored differently and represented by arrows drawn approximately to scale. The *cop* gene cluster characterized by reference 41 is highlighted with orange color. The *attL* and *attR* sites are present near tRNA sites in red. Shown below are the nucleotide sequence flanking the genomic island (blue); att sites *attL, attR,* and *attB (*red); and chromosomal DNA (black) in *X. euvesicatoria* pv. *euvesicatoria* donor strain XvP26 and recipient strain X82-8.

Chromosomal CuR was first identified in another *X. euvesicatoria* pathovar, *X. euvesicatoria* pv. *euvesicatoria* strain XvP26 (40, 41). However, in all *X. euvesicatoria* pv. *euvesicatoria* strains examined in this study, except XvP26, copper resistance was plasmid borne (Fig. 4A; Table S4). Basim et al. (40) demonstrated the horizontal transfer of chromosomal *cop* genes present in XvP26 strain through conjugation. To understand the integration mechanism of chromosomal *cop* gene transfer, we sequenced transconjugant strains from that study and a replicate conjugation experiment. A total of six transconjugants were sequenced. The whole-genome characteristics of sequenced transconjugants are provided in Table S5. The results showed the transfer of a 105 kb chromosomal region to the *X. euvesicatoria* pv. *euvesicatoria* recipient strain X82-8, which integrated near tRNA^leu^ (TAA) and is flanked by 17 bp direct repeats (CTCGTTTCCCGCTCCAA) representing *attL* and *attR* sites (Fig. 4B). The results show the movement of chromosomal-encoded CuR from one strain to another through an ICE in *X. euvesicatoria* pv. *euvesicatoria*. Upon analyzing the genomic island present in *X. euvesicatoria* pv. *perforans*, it appears to be an integrative genomic island as it lacks the complete machinery required for its self-mobility and may require helper prophages or conjugative elements.

### Comparative analysis of chromosomal and plasmid-borne CuR

**Fig 5:**
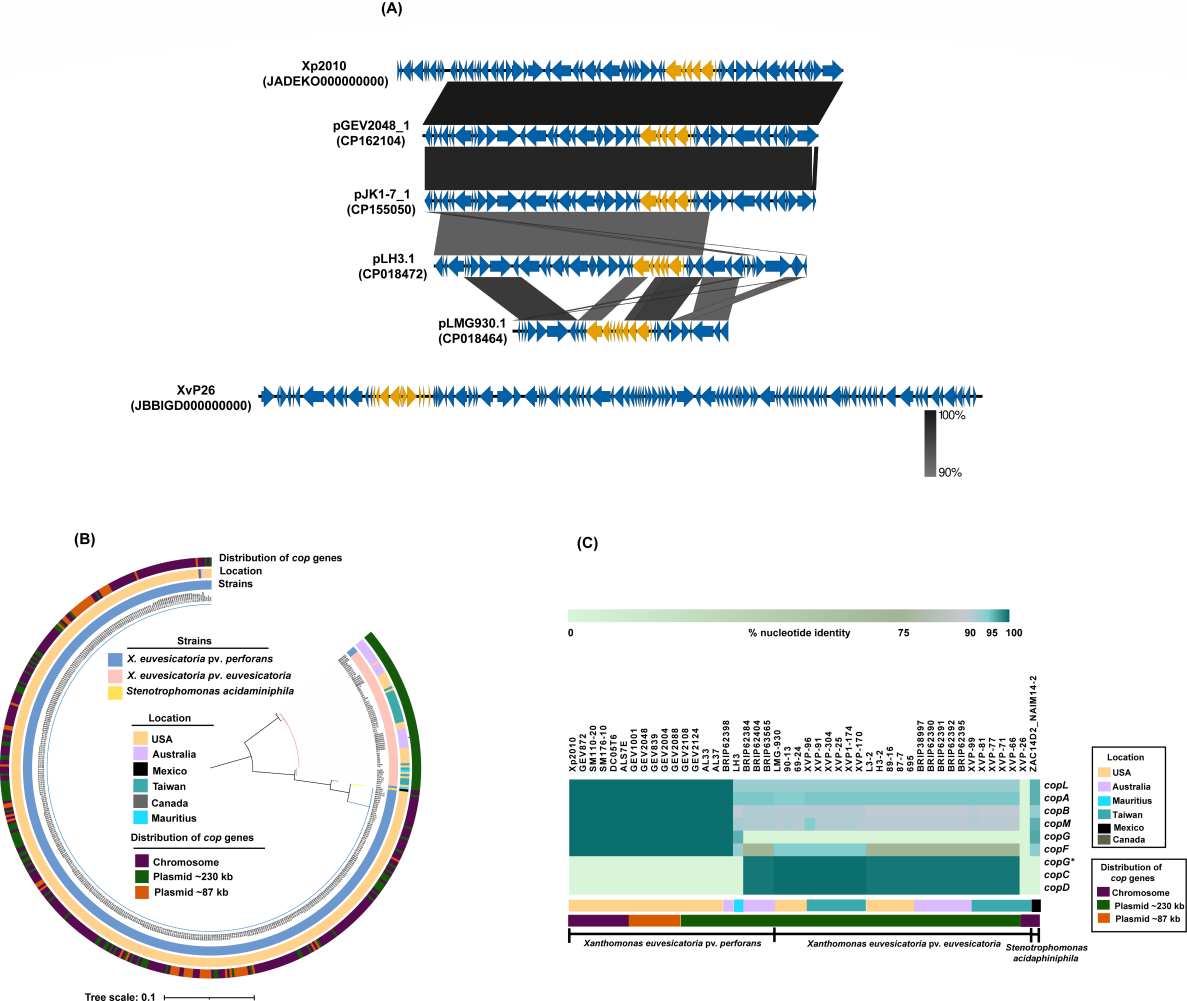
Comparative analysis of genomic islands and copper genes. (**A**) Comparison showing similarity of the genomic island present in *X. euvesicatoria* pv. *perforans* strain Xp2010 with the homologous region in copper plasmids pGEV2048_1 (CP162104), pJK1-7_1 (CP155050), and pLH3.1 (CP018472) in *X. euvesicatoria* pv. *perforans*, as well as pLMG930.1 (CP018464) in *X. euvesicatoria* pv. *euvesicatoria* and the genomic island present in *X. euvesicatoria* pv. *euvesicatoria* strain XvP26. Genes are represented by arrows, and the percentage identity is indicated by connecting links between genes. The *cop* genes are highlighted in orange color. (**B**) Maximum-likelihood phylogenetic tree constructed based on the concatenated *copL*, *copA*, *copB*, *copM*, *copG*, *copF*, *copC*, and *copD* genes among *X. euvesicatoria* pv. *perforans*, *X. euvesicatoria* pv. *euvesicatoria,* and *Stenotrophomonas acidaminiphila* strains. The strain names, their geographic locations, and distribution of *cop* genes on the chromosome or plasmid are indicated by different colors. (**C**) A comparison of *cop operon* (*copLABMGF*) in *X. euvesicatoria* pv. *perforans s*train Xp2010 as a reference with representative strains of *X. euvesicatoria* pv. *perforans and X. euvesicatoria* pv. *euvesicatoria* strains from each clade in Fig. 5B, where *cop* genes are present either on a genomic island or a plasmid. For *copC*, *copD,* and copG* genes that are absent in Xp2010, the identity is determined with respect to *cop* genes present on the plasmid from *X. euvesicatoria* pv. *euvesicatoria* strain LMG930.

Bibi et al. (42) showed the comparative analysis of chromosomal and plasmid-borne CuR using the *X. euvesicatoria* pv. *perforans* strain Xp2010 genomic island and a copper plasmid present in *X. euvesicatoria* pv. *perforans* strain LH3 from Mauritius. The analysis revealed high similarity between the two, except for the absence of the phage region in LH3, gene rearrangements, and the presence of additional heavy metal resistance genes in Xp2010 genomic island. Interestingly, we found that the Xp2010 genomic island is highly identical to copper plasmids from *X. euvesicatoria* pv. *perforans* strains from Florida. Comparative analysis indicated that a 57,249-bp core region of the genomic island is 99.98% identical with 100% query coverage to a region of the 87 kb pGEV2048_1 (CP162104), whereas it shows 97.69% identity with 99% query coverage to pJK1-7_1 (CP155050) and 93.27% identity with 94% query coverage to pLH3.1 (CP018472) (Fig. 5A). While it appears that a 57 kb region of plasmid integrated into the chromosome or vice versa through transposase-mediated transposition, the exact mechanism remains undetermined. Notably, in *X. euvesicatoria* pv. *perforans* strain AL1, which lacks the *cop* genes, a part of the phage region from the genomic island was present (54% query coverage and 90.93% identity) (Fig. S4). Additionally, other strains that lack the *cop* genes, such as those from Turkey, Canada, Alabama, and Australia, also contain phage genes, similar to the AL1 strain, in the same region where the genomic island is found in strains that possess the genomic island. The Xp2010 genomic island exhibited lower similarity (i.e., 95.15% identity with 48% query coverage) to the pLMG930.1 (CP018464) plasmid from *X. euvesicatoria* pv. *euvesicatoria* strain LMG930. Furthermore, the genomic island in *X. euvesicatoria* pv. *euvesicatoria* XvP26 showed no similarity to the genomic island in Xp2010 or pLMG930, suggesting independent acquisition (Fig. 5A).

The variation between pGEV2048_1, pJK1-7_1, and pLH3.1 plasmids prompted us to investigate the identity of *cop* genes in *X. euvesicatoria* pv. *perforans* strains (Fig. 5B and C). We observed that *cop* genes present either on the genomic island or plasmid exhibited 100% nucleotide identity with *copLABMGF* from Xp2010 in all strains from the USA (Florida, Alabama, Ohio) (Table S6). A phylogeny constructed based on the *cop* genes grouped the strains based on the conservation of nucleotide sequences of these genes (Fig. 5B). A strain from Canada (DC05T6) also has *cop* genes in the genomic island that were 100% identical to those from the USA. One strain from Australia (BRIP6298) has *cop* genes on the plasmid that were 100% identical to those from the USA. However, for other strains from Australia and Mauritius, with *cop* genes present on the plasmid, nucleotide identity varied between 90% and 98% (Fig. 5C; Table S6). In the core genome tree- and *cop* genes-based phylogeny, strains with identical *cop* genes grouped with U.S. strains (Fig. 5B). The *cop* operon (*copLABMGCDF*) in most *X. euvesicatoria* pv. *perforans* strains from Australia was more like that found in *X. euvesicatoria* pv. *euvesicatoria* strains. In contrast to *X. euvesicatoria* pv. *perforans,* in *X. euvesicatoria* pv. *euvesicatoria,* the *cop* operon was exclusively plasmid borne, with the exception of XvP26. The *cop* genes in XvP26 did not show identity with any *cop* genes from *X. euvesicatoria* pv. *perforans* nor *X. euvesicatoria* pv. *euvesicatoria*. Furthermore, as previously shown by Bibi et al. (42), the island in Xp2010 is related to a genomic island from *Stenotrophomonas*. We compared *cop* genes present in a genomic island from *S. acidaminiphila* strain ZAC14D2_NAIM14-2 (CP012900) to those in *X. euvesicatoria* pv. *perforans* strains from the USA and found higher identity compared to *cop* genes present in pLH3.1 and pLMG930.1 (Fig. 5C).

## DISCUSSION

Horizontal gene transfer (HGT), mediated by MGEs, significantly contributes to bacterial diversification and adaptation (48, 49). HGT of CuR in *Xanthomonas* occurs frequently through a large plasmid-carrying *cop* genes (25, 28, 50, 51). Recently, there have been an increasing number of reports of chromosomal CuR, indicating that resistance is not limited to plasmids. Chromosomal CuR acquired through genomic islands has been reported in *X. arboricola* pv. *juglandis*, *X. euvesicatoria* pv. *perforans,* and *X. campestris* pv. *campestris* (42, 43, 52). The widespread occurrence of CuR in *X. euvesicatoria* pv. *perforans* prompted us to look for its acquisition mechanism. An extensive collection of *X. euvesicatoria* pv. *perforans* genomes from various geographical areas was analyzed to determine the distribution of plasmid and chromosomal CuR. Chromosomal CuR was found to be widespread, identified in 323 genomes, compared to plasmid-borne resistance, which was found in 102 genomes. Many of the strains with chromosomal resistance were collected from Florida and Ohio, USA. It appears that once established within the population, chromosomal resistance persists through vertical transmission, which may be beneficial for *X. euvesicatoria* pv. *perforans,* given the consistent use of copper-based products for disease management on tomatoes in Florida. Carrying chromosomal CuR is expected to eliminate the energy cost associated with maintaining and replicating large copper plasmids. Moreover, without selection pressure, plasmid-borne resistance genes can be lost through plasmid loss within the population (53, 54). A study by Subedi et al. (55) revealed that copper-sensitive *X. euvesicatoria* pv. *perforans* strains were prevalent in a pepper farm where copper bactericides were not used. In our analysis, strains from Alabama (USA), Turkey, Canada, and Italy lack both a copper plasmid and a genomic island. However, data regarding the sensitivity or resistance of these strains are unavailable, and how frequently copper sprays are used to manage BST in those areas is unknown.

Surprisingly, we identified strains that carried both chromosomal and plasmid-borne CuR. Our results showed that plasmids confer slightly higher copper tolerance than chromosomal-encoded CuR alone. This increased tolerance could be due to the multiple copies of the plasmid per cell, resulting in increased expression of *cop* genes. Multi-copy resistance may provide an evolutionary advantage under strong selection from copper treatments, even though plasmids may impose greater fitness cost in the absence of copper. We speculate that there has been a transition in *X. euvesicatoria* pv. *perforans* populations in the USA to chromosomal CuR, but some strains acquired an additional copy of *cop* operon via plasmid in response to heavy copper usage.

Although most strains contained the chromosomal copper genomic island at the same location in the genome, the genes present within this island showed evidence of a dynamic nature, undergoing further gene deletions, mutation, and recombination. Different variants of the island are present within and between different phylogenetic groups, suggesting that the island has evolved since its initial acquisition. Bibi et al. (42) showed that the genomic island is flanked by phage genes at one end. We found this region constitutes the variable part of the island undergoing gene deletions. Phage regions are dynamic zones within the genome where genetic variation is high and prone to deletions to minimize the metabolic burden required to replicate phage genes (56). In contrast, the core part of the island contains genes critical for bacterial adaptation and survival. Nucleotide variation in the core region is consistent with homologous recombination affecting genes related to RND efflux system, TonB-dependent receptors, and heavy metal-associated genes that may be required to withstand antimicrobial compounds.

Previous analyses of genomic variation in populations of *X. euvesicatoria* pv. *perforans* found evidence of recombinant regions in the core genome that was consistent with horizontal gene transfer from *X. euvesicatoria* pv. *euvesicatoria* (57–59). However, our comparative analysis of chromosomal CuR in *X. euvesicatoria* pv. *perforans* and *X. euvesicatoria* pv. *euvesicatoria* revealed distinct origins of CuR. The core part of the *X. euvesicatoria* pv. *perforans* island (57 kb) was highly identical to the region on the copper plasmid present in USA strains. The pLH3.1 copper plasmid from *X. euvesicatoria* pv. *perforans* strain from Mauritius and pLMG930 from *X euvesicatoria* pv. *euvesicatoria* contained genetically distinct *cop* genes. In contrast, the chromosomal island characterized in *X. euvesicatoria* pv. *euvesicatoria* strain XvP26 showed no significant similarity with the island present in Xp2010. To date, chromosomal copper resistance in *X. euvesicatoria* pv. *euvesicatoria* has been found only in strain XvP26, while for other copper-tolerant *X. euvesicatoria* pv. *euvesicatoria* strains, the *cop* operon was located on a plasmid. In addition, the genetic variation in the genomic island suggests independent acquisitions of copper resistance in *X. euvesicatoria* pv. *perforans* and *X. euvesicatoria* pv. *euvesicatoria*. While the 57 kb region of the island that is highly identical to the copper plasmid in *X. euvesicatoria* pv. *perforans* from the USA suggests a shared origin, the likelihood of integration from plasmid to chromosome or vice versa remains undetermined. The mobility of the genomic island in *X. euvesicatoria* pv. *perforans,* whether through phages or plasmid integration, is unclear. On the other hand, the chromosomal island in *X. euvesicatoria* pv. *euvesicatoria* is categorized as an ICE and possesses machinery required for self-transfer. We were able to demonstrate the mobility of this island suggesting the potential for horizontal spread of CuR through genomic islands in *X. euvesicatoria* pv. *euvesicatoria*.

Within the *cop* operon, the phylogenetic analysis based on *copLAB* gene sequences has revealed a common origin of CuR but independent exchange of *cop* genes among *Xanthomonas* species (30, 60). In our study, the *cop* operon in *X. euvesicatoria* pv. *perforans* strains contains *copLABMGF* genes, while *X. euvesicatoria* pv. *euvesicatoria* has copLABMG*CDF (copG*CD similar to *copGCD* genes characterized in *X. citri* pv. *citri* [29]). Interestingly, the *cop* operon (*copLABMGF*) was highly identical (100% identity for all genes) in the island and plasmid in all *X. euvesicatoria* pv. *perforans* strains from the USA, while variability in sequence identity (between 74% and 95%) was noted when compared with *X. euvesicatoria* pv. *perforans* from Australia and Mauritius and *X. euvesicatoria* pv. *euvesicatoria* strains. This variation could be attributed to distinct tomato breeding and production systems in the USA compared to locations such as Australia and Mauritius. Additionally, the exchange of transplants produced in southern states to northern regions of the USA increases the possibility of shared plasmid acquisition or chromosomal movement. The CuR island found in *X. euvesicatoria* pv. *perforans* has been previously hypothesized to be acquired from *Stenotrophomonas* (42). We could not find *Stenotrophomonas cop* genes with 100% identity, although there is high identity (between 94% and 97%) with *cop* genes from *S. acidaminiphila*.

In conclusion, our findings show different patterns of evolution of copper resistance in *X euvesicatoria* pv. *perforans* and *X euvesicatoria* pv. *euvesicatoria*. The role of MGEs in spreading CuR reinforces the need for the development of new strategies for BST control, alongside a deep understanding of resistance mechanisms, to deploy effective disease management approaches.

## MATERIALS AND METHODS

### Copper resistance distribution and phylogenetic analysis

All computational analysis was performed on the University of Florida HiPerGator supercomputers. Plasmid prediction was done using the MOB-suite tool (v3.1.0) (46). For chromosomal CuR prediction, Blast analysis for the genomic island as a query was performed using BLASTN (v2.15.0+). The reported genomic island by Bibi et al. (42), which carries CuR genes, was used as a query along with 5 kb upstream and downstream regions. For analysis, 452 available genomes of *X. euvesicatoria* pv. *perforans* and 124 *X*. *euvesicatoria* pv. *euvesicatoria* were retrieved from NCBI (https://www.ncbi.nlm.nih.gov/). The genomes were annotated using Prokka (v1.14.6) (61), and Roary (v1.007002) (62) was then used to obtain a core gene alignment. A total of 2,981 core genes were identified as present in more than 99% of the *X. euvesicatoria* pv. *perforans* strains, and the alignment length was 2.99 Mbp. In the case of *X. euvesicatoria* pv. *euvesicatoria* genomes, a total of 3,872 core genes were identified with an alignment length of 4.03 Mbp. The core gene alignment file obtained from Roary was used as an input for PhyML (v3.3.20220408) (63) to generate a phylogenetic tree. Furthermore, recombination sites were detected using ClonalFrameML (64) and masked with maskrc-svg (https://github.com/kwongj/maskrc-svg). The masked file was then used to construct final phylogenetic tree using Phyml (v3.3.20220408) with 500 bootstraps, and the final tree was visualized with iTOL software (v) (65). Clustering analysis was done using fastbaps (47).

### Determination of CuR tolerance

Strains carrying either chromosomal or plasmid-borne copper resistance, or both were selected. To assess the level of CuR, growth was assessed on nutrient agar plates amended with different concentrations of cupric sulphate (CuSO_4_·5H_2_O). Strains were grown overnight on nutrient agar amended with 20 ppm copper to induce CuR genes. The growth was suspended in sterile tap water and diluted to 10^8^ cfu/mL, and then serial dilutions were made up to 10^1^ cfu/mL. Ten microliters of each dilution was spotted on nutrient agar plates without and with different concentrations of copper (200, 250, 300, 350, and 400 ppm). The plates were then incubated at 28°C, and growth was examined after 48 h. The experiment was performed three times. The copper-sensitive *X. euvesicatoria* pv. *perforans* strain 91-118 was used as a negative control.

### Plasmid extraction

The extraction of plasmids was performed as described by Kado and Liu with some modifications (66). The detection of extracted plasmids was performed by electrophoresis as described previously (51).

### Comparison of genes present in genomic island

Sixty genes present in the genomic island identified in Xp2010 were used as a query for BLASTN (v2.15.0) against the genomes of strains, in which the genomic island was assembled in a single contig. The cutoff for percent nucleotide identity was 70%, and gene length coverage was 40%. A heatmap was generated based on the percent nucleotide identity using GENE-E tool (https://software.broadinstitute.org/GENE-E/). For phylogenetic analysis of core region of genomic island, the core region was extracted from all representative strains and aligned with MAFFT (v7.520) (67), and then a maximum likelihood tree was built using PhyML (v3.3.20220408). Tree was visualized with iTOL software. Recombination in the core region was detected using ClonalFrameML (v1.12) (64).

### Conjugation assay

A conjugation experiment for *X. euvesicatoria* pv. *euvesicatoria* strains was performed as previously described in reference 40. Briefly, donor strain XvP26 (resistant to copper and streptomycin) and recipient strain X82-8P (Pig^-^ mutant of strain X82-8) (copper sensitive, resistant to nalidixic acid and rifamycin) were grown overnight at 28°C on NA containing appropriate antimicrobials. Antimicrobial agents were used at final concentrations: cupric sulfate (CuSO_4_·5H_2_O) 200 µg mL^−1^, streptomycin 50 µg mL^−1^, nalidixic acid 50 µg mL^−1^, and rifamycin 100 µg mL^−1^. Bacteria were collected from plates, suspended in 1 mL sterile tap water, and washed twice by centrifugation, and pellets were resuspended in sterile water. Both donor and recipient were then mixed in equal proportion (10^9^ cfu/mL), and 10 µL spotted on nutrient yeast glycerol agar (NYGA). Plates were incubated for 48 h. Then, cells were scraped from the plate and resuspended in 1 mL sterile water, and 500 µL total cell suspension was plated on five plates (100 µL per plate) containing selective media with copper, nalidixic acid, and rifamycin. Colonies obtained were restreaked on the selective media again to confirm the stability.

### Genome sequencing

Whole-genome sequencing of *X. euvesicatoria* pv. *euvesicatoria* transconjugants from the study by Basim et al. (40) and from the present study was done to confirm the integration sites in *X. euvesicatoria* pv. *euvesicatoria*. DNA was extracted from the overnight grown cultures using the Wizard Genomic DNA Purification Kit (Promega, Madison, WI). DNA samples were sent to the SeqCenter (Pittsburgh, PA, USA) for Illumina sequencing. Adapters were removed from the raw reads using Trim Galore (v0.6.10) with default parameters (68). Reads were assembled using SPAdes (v3.10.1) with –careful parameters and k-mer length 21, 33, 55, 77, 99, and 127 (69). Contigs smaller than 500 bp and k-mer coverage 2.0 were filtered out. Draft assemblies were polished using Pilon (v1.24) with the default parameters (70). The completeness and contamination of assembled genomes were checked using CheckM (v1.1.2) (71). Assembled genomes were submitted to NCBI GenBank database under BioProject number PRJNA1088443 and annotated using NCBI PGAP pipeline. Accession numbers for each genome are provided in Table S3. The ICE attachment sites in *X. euvesicatoria* pv. *euvesicatoria* transconjugants were manually searched by comparing donor XvP26 and recipient X82-8 genome. For Nanopore sequencing of strains GEV2048 and GEV1001, extracted DNA was sent for sequencing to Plasmidsaurus Inc. Raw reads were filtered using Filtlong (v.0.2.0) (72) excluding reads less than 5,000 bp. Filtered reads were assembled using Flye (v2.9.3-b1797) (73) and Unicycler (v0.4.8) (74). Assembled files were polished using Pilon and assembled files were submitted to NCBI GenBank database and annotated using NCBI PGAP pipeline. The accession number for genomes is provided in Table S3.

### Comparative analysis of chromosomal and plasmid CuR

The comparative analysis of chromosomal and plasmid-borne copper resistance was performed using Easyfig tool (75). For *cop* genes analysis, the *cop* operon (*copLABMGF*) present in the *X. euvesicatoria* pv. *perforans* strain Xp2010 genomic island was taken as query to compare gene identity with other *X. euvesicatoria* pv. *perforans* and *X. euvesicatoria* pv. *euvesicatoria* genomes using BLASTN (v2.15.0+). A phylogeny of *cop* genes was constructed by concatenating all the *cop* genes, followed by alignment using MAFFT and tree construction using PhyML.

## Data Availability

Assembled whole-genome sequences were deposited in the NCBI GenBank database under BioProject number PRJNA1088443, and accession numbers are provided in Tables S1, S4, and S5.

## SUPPLEMENTAL MATERIAL

Supplemental Figures msystems.01427-24-s0001.docx Figures S1 to S4.

Supplemental Tables msystems.01427-24-s0002.xlsx Tables S1 to S6.

ASM does not own the copyrights to Supplemental Material that may be linked to, or accessed through, an article. The authors have granted ASM a non-exclusive, world-wide license to publish the Supplemental Material files. Please contact the corresponding author directly for reuse.
