# Supplementary material for: Evolution of copper resistance in Xanthomonas euvesicatoria pv. perforans population
Source: mSystems. 2024 Nov 25;9(12):e01427-24. doi: 10.1128/msystems.01427-24 (PMC11651105; doi:10.1128/msystems.01427-24)
Supplement: Supplemental Figures — Figures S1 to S4. [file msystems.01427-24-s0001.docx]

**Supplementary Figures:**


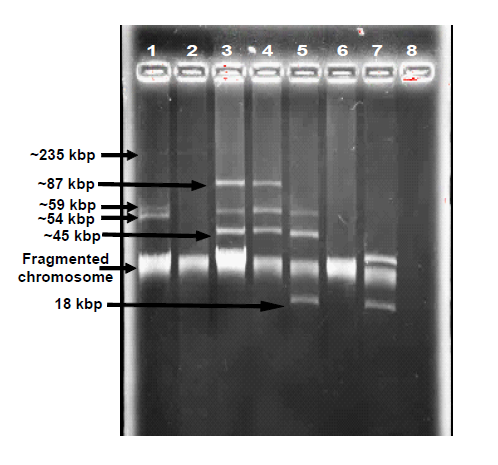


Fig. S1: Plasmid profile of *X. euvesicatoria* pv. *perforans* strains: 1. JK22-3, 2. GEV2010, 3. GEV1001, 4. GEV2048, 5. GEV915, 6. Xp2010, 7. 91-118. Strains JK22-3 and GEV2010 have a large (~235 kb) copper plasmid, whereas GEV1001 and GEV2048 have a small ~87 kb copper plasmid. Strains GEV915 and Xp2010 have only chromosomal CuR and contain no copper plasmid, while 91-118 is copper sensitive.


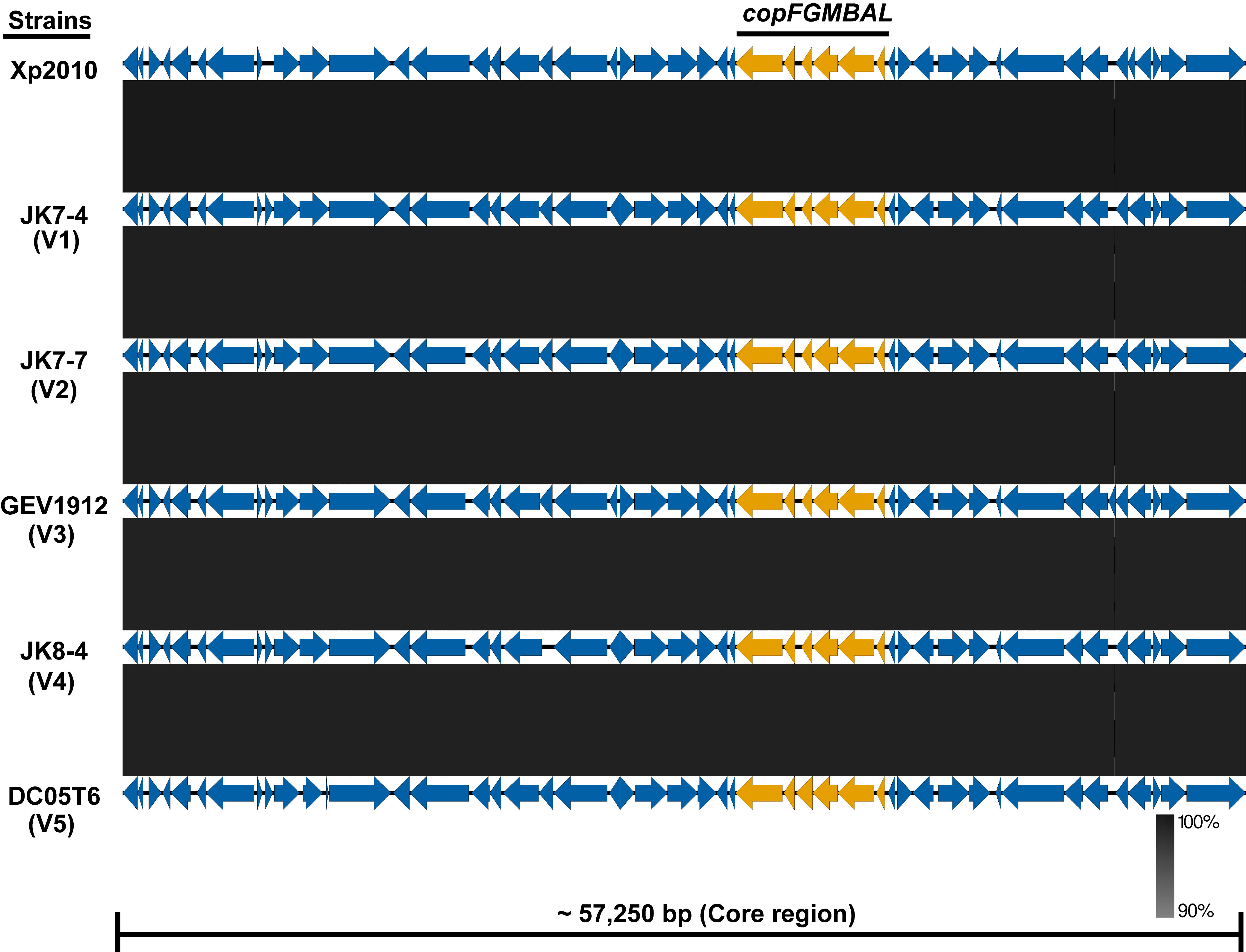


Fig. S2: Gene synteny of the core region of the genomic island is conserved in selected representative strains from each variant of the genomic island. Genes are represented by arrows and the percentage identity is indicated by connecting links between genes. The *cop* operon is highlighted in the orange.


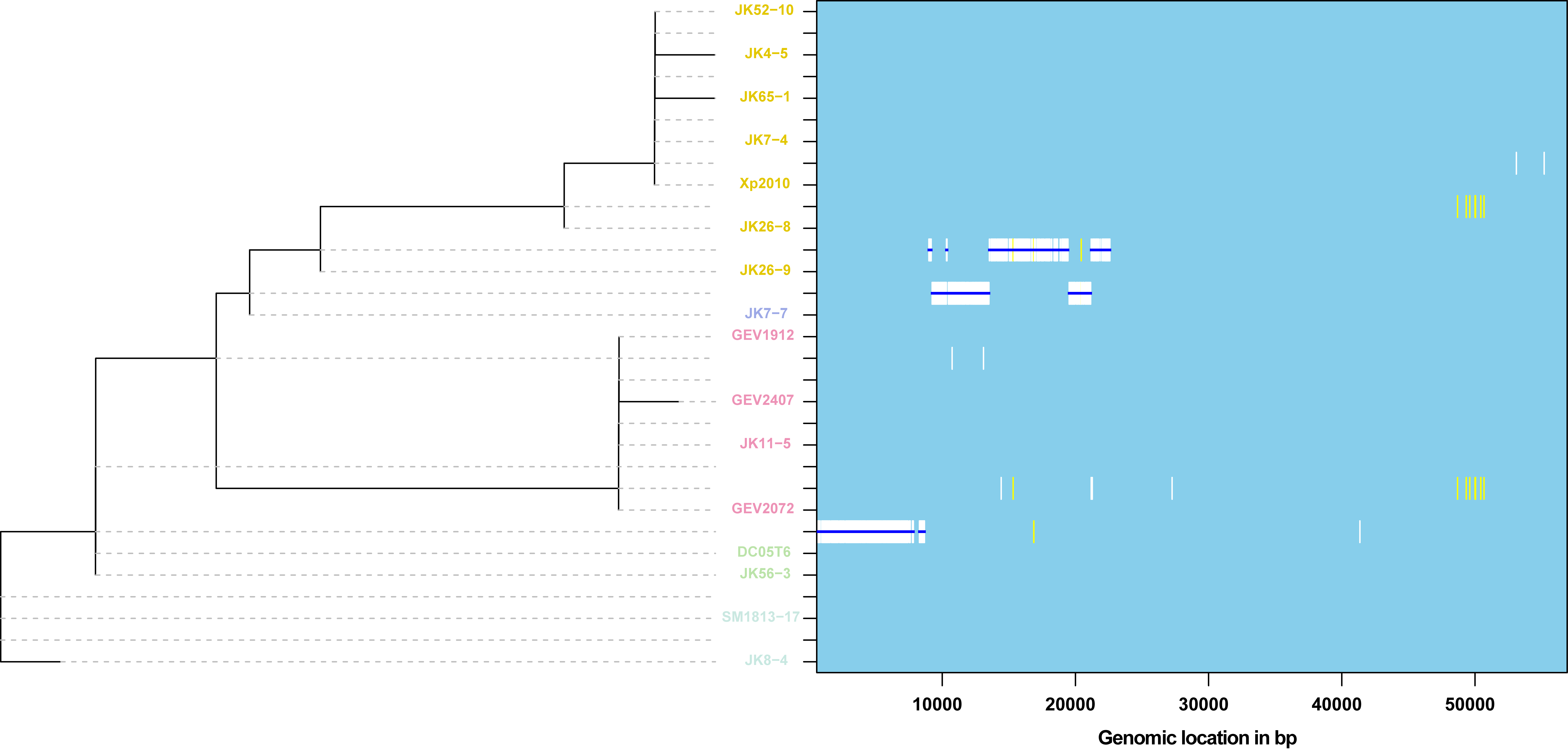


Fig. S3: ClonalFrameML analysis of the core region of the genomic island in representative strains from each variant of the genomic island. The tree on the left is the clonal genealogy inferred by ClonalFrameML after accounting the effect of recombination. For each branch of the tree, a corresponding row in the heatmap on the right, highlights the identified polymorphisms. Dark blue horizontal bars indicate the sites where recombination events have been inferred near the genes INP48_22240-INP48_22325, where variability in nucleotide identity is noted as shown in Fig. 3A. The strain names highlighted in different colors refers to the different variants of the genomic island identified from the core region phylogeny in Fig. 3B. Another color indicates level of homoplasy: white lines indicate no homoplasy whereas yellow represents homoplasy.


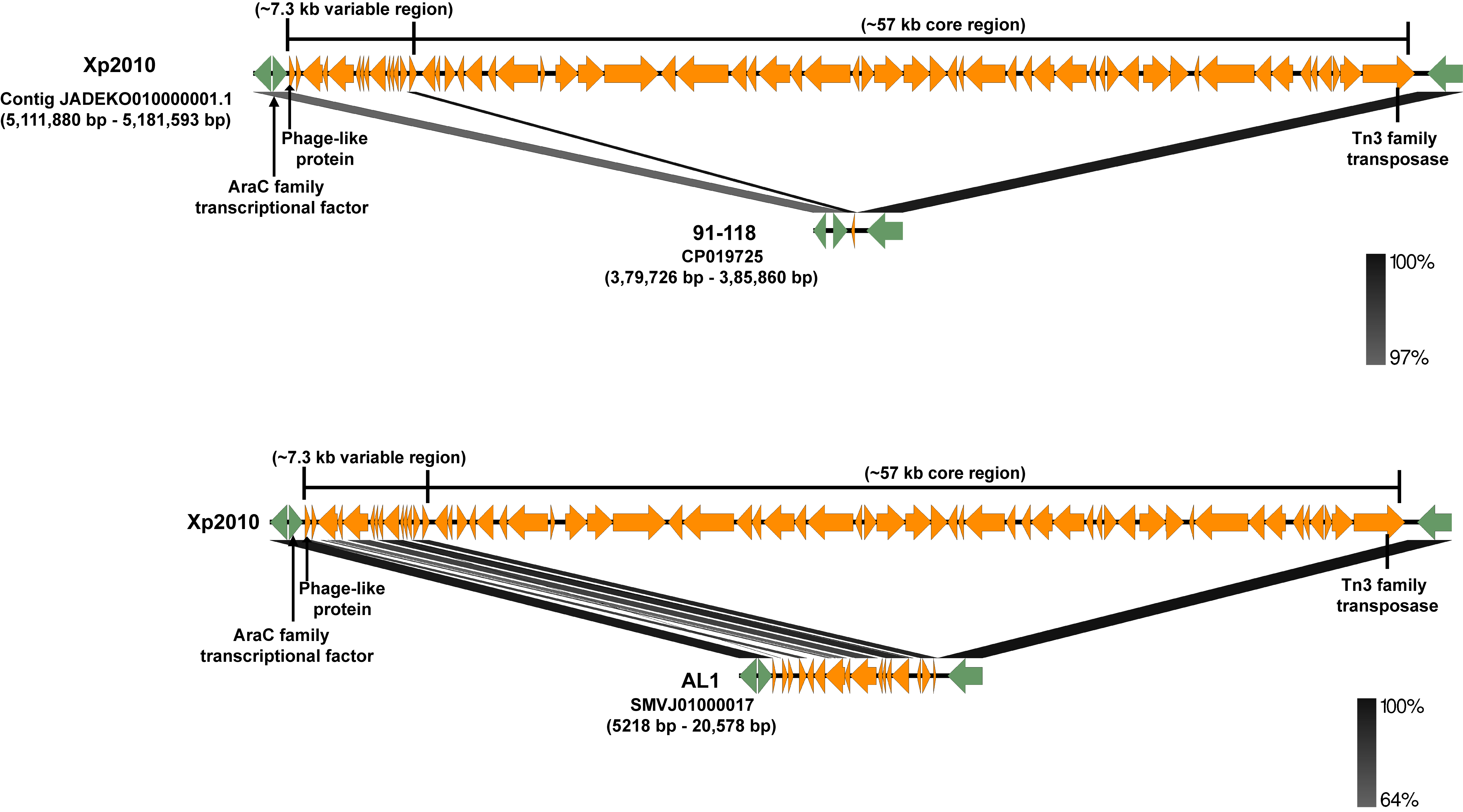


Fig. S4: Comparative analysis of *X. euvesicatoria* pv. *perforans* strain Xp2010, which carries a genomic island, with the *X. euvesicatoria* pv. *perforans* strains 91-118 and AL1 which lack the genomic island. Genes are represented by arrows and the percentage identity is indicated by connecting links between genes. Orange arrows represent genes within the genomic island, while green arrows represent flanking genes present in both strains with and without the genomic island. The coordinates of the complete region in the genomes are listed below the strain name along with the contig number. The direction of the AL1 region was reversed for comparison.
